# Phosphorylation-mediated interaction between human E26 transcription factor 1 and specific protein 1 is required for tumor cell migration: Ets1 and Sp1 interact to promote SW480 migration

**DOI:** 10.3724/abbs.2022148

**Published:** 2022-10-27

**Authors:** Xianhui Wen, Xingsheng Sun, Zheyuan Ou, Jun Jiang, Qingmei Chen, Xirong He, Zhangsheng Hu, Han Qiao, Kuan Zhou, Xin Li, Yiqun Deng, Jikai Wen

**Affiliations:** 1 Guangdong Provincial Key Laboratory of Protein Function and Regulation in Agricultural Organisms College of Life Sciences South China Agricultural University Guangzhou 510642 China; 2 Guangdong Laboratory for Lingnan Modern Agriculture South China Agricultural University Guangzhou 510642 China; 3 Key Laboratory of Zoonosis of Ministry of Agriculture and Rural Affairs South China Agricultural University Guangzhou 510642 China

**Keywords:** protein interactions, Ets1, Sp1, PP2, Dasatinib, colon cancer

## Abstract

Transcription factors, human E26 transcription factor 1 (Ets1) and specific protein 1 (Sp1), are known to induce gene expression in tumorigenicity. High Ets1 expression is often associated with colorectal tumorigenesis. In this study, we discover that metastasis and clone formation in SW480 cells mainly depend on the direct interaction between Ets1 and Sp1 instead of high Ets1 expression. The interaction domains are further addressed to be the segment at Sp1(626-708) and the segment at Ets1(244-331). In addition, the phosphorylation inhibition of Ets1 at Tyr283 by either downregulation of Src kinase or Src family inhibitor treatment decreases the interaction between Sp1 and Ets1 and suppresses SW480 migration. Either administration or overexpression of the peptides harboring the interaction segment strongly inhibits the colony formation and migration of SW480 cells. Our findings suggest that the interaction between Ets1 and Sp1 rather than Ets1 alone promotes transformation in SW480 cells and provide new insight into the Ets1 and Sp1 interaction as an antitumour target in SW480 cells.

## Introduction

As a member of the ETS domain transcription factor subfamily, Ets1 is widely characterized as an oncoprotein transcription factor due to its high expression level linked to poor survival in many carcinomas, including colorectal, breast, cervical, gastric, lung, oral and ovarian cancer [ 1– 10] . Additionally, Ets1 knockdown impairs tumor growth in breast and melanoma cancer [ 11, 12] and the progression of proliferative, metastatic, and invasive gastric cancer [13]. The C-terminal Exon VII domain of Ets1 is often integrated by upstream signals to regulate its protein stability. For example, Src stabilizes Ets1 by phosphorylating Tyr283 in the exon VII domain of Ets1 [11].

The Src protein, a member of the SRC family, is a proto-oncogene tyrosine-protein kinase [ 14, 15] . Recently, a study showed that inhibition of the Src/Ets1 pathway, which results in downregulation of Ets1 protein level, may provide treatment for cisplatin-resistant head and neck squamous cell carcinoma [16]. Many strategies which target the reduction of Ets1 protein level as a tumor treatment option have been studied. However, there are few studies on the regulation of tumorigenesis by affecting other activities of Ets1.

Ets1 often participates in the transcriptional regulation of downstream target genes with other regulatory factors. Ets1 interacts with other transcription factors to promote the regulation of downstream target genes, such as *Sp1*. Sp1 is also a transcription factor and a housekeeping gene protein, so it can participate in a variety of cell life activities, including proliferation, differentiation, DNA damage response, apoptosis, and angiogenesis [ 17– 20] . In addition, as a cancer-promoting factor, Sp1 is overexpressed in thyroid cancer, gastric cancer, pancreatic cancer, glioma, and breast cancer, leading to poor prognosis [ 21– 25] . Since both Ets1 and Sp1 are involved in the process of tumorigenesis and can synergistically induce gene expression [ 26– 29] , it is interesting to explore their interaction in tumorigenesis.

Ets1, Sp1 and Src all regulate cell progression in colorectal cancer [ 30– 34] , and additionally, both Ets1 and Src are highly expressed in colorectal cancer [ 10, 33, 34] , implying that there may be reciprocal links between Ets1, Sp1 and Src in the development of colorectal cancer. In the present study, we studied the interaction between these two transcription factors using SW480 colorectal cancer cells. We found that metastasis and clone formation in SW480 cells depended on the direct interaction between Ets1 and Sp1, and the domains involved in this interaction were further addressed to be the segment at Sp1(626-708) and the segment at Ets1(244-331), respectively. Furthermore, Src kinase phosphorylated Tyr283 , an amino acid residue in the exon VII domain of Ets1, which promoted this interaction and increased SW480 migration. Our findings suggested that the interaction between Ets1 and Sp1 rather than the Ets1 protein level alone affects the transformation in SW480 cells.

## Materials and Methods

### Plasmids

The full-length complementary DNAs (cDNAs) encoding human Ets1 and Sp1 were cloned into pET28a or pcDNA3.1 vectors for prokaryotic or mammalian cell expression studies. For mammalian cell transfection, deletion mutants of *SP1* and *ETS1* and point mutations of *ETS1* were prepared by overlapping PCR and verified by DNA sequencing. For TAT-Ets1(244-331)-GFP, the DNA fragments encoding the TAT (YGRKKRRQRRR) peptide and Ets1(244-331) sequences were synthesized and inserted into the pET28a expression vector at the sites of *Nco*I and *Bam*HI. The EGFP sequences were sequentially inserted into the vector at the sites of *Bam*HI and *Xho*I. For TAT-Sp1(626-708)-mCherry, the synthetic TAT-Sp1(626-708) peptide was inserted into the pET28a expression vector at the sites of *Nco*I and *Bam*HI. Next, the mCherry sequences were inserted into the sites of *Bam*HI and *Xho*I. For the Co-IP assay, Flag, HA, and GST epitope tags were fused to the C-terminus of Sp1 and Ets1. For the BIFC assay, Ets1-Vc155 and Sp1-Vn173 and the mutant plasmids were cloned into pcDNA4.0 vectors.

### Cell culture

SW480 (CCL-228; ATCC, Manassas, USA), WM793 (CRL-2806; ATCC), and HEK293T (CRL-11268; ATCC) cell lines were preserved in our laboratory. All the cell lines were regularly tested and confirmed to be negative for mycoplasma contamination. The cells were cultured in high D-glucose DMEM (Invitrogen, Carlsbad, USA) supplemented with 10% FBS (BI, Gottingen, Germany) at 37ºC in a CO _2_ incubator (Thermo Fisher, Waltham, USA).

### CCK-8 assay

The cytotoxic effects of dasatinib and PP2 were determined in the indicated cell lines using a cell counting kit-8 (CCK-8) assay kit (Sangon Biotech, Shanghai, China). Briefly, a total of 5000 cells were seeded in each well of a 96-well plate. After treatment with 0, 1 and 5 μM dasatinib (Selleck, Shanghai, China) or 0, 1, 5 and 10 μM PP2 (Selleck) for 24 h at 37ºC, 10 μL CCK-8 solution (0.5 mg/mL) was added to each well, and the cells were incubated for 1 h at 37ºC. The optical density was determined at 450 nm with a microplate reader (Promega, Madison, USA).

### Western blot analysis

The total cell lysates were prepared by using cold radioimmunoprecipitation assay (RIPA) lysis buffer (50 mM Tris-HCl, pH 7.8, 150 mM NaCl, 1% Triton X-100, pH 7.8) containing protease and phosphatase inhibitors for 30 min on ice and then centrifuged for 10 min at 14,000 *g*. The lysates were subjected to sodium dodecyl sulfate-polyacrylamide gel electrophoresis (SDS-PAGE) and transferred to polyvinylidene fluoride membranes (Millipore, Billerica, USA). After being blocked, membranes were incubated overnight at 4ºC with indicated primary antibodies diluted at 1:1000 in primary antibody diluent (Beyotime Biotechnology, Shanghai, China), followed by incubation with the corresponding secondary antibodies conjugated to HRP diluted at 1:5000 in TBST buffer (200 mM Tris base, pH 8.0, 1500 mM NaCl and 0.1% Tween-20) for 1 h at room temperature. Enhanced chemiluminescence (ECL) mix (Genview, Beijing, China) was used to visualize the protein bands in a dark room. The antibodies used are shown in Table 1.

**Table:** **Table 1** Antibodies used in the current study

Antigen	Species	Clonality	Clone	Catalog	Manufacturer
Ets1	Rabbit	Monoclonal	D8O8A	#14069	Cell Signal Technology (Beverly, USA)
Sp1	Rabbit	Polyclonal		ab13370	Abcam (London, UK)
Phospho-tyrosine mouse mAb	Mouse	Monoclonal	P-Tyr-100	5465	Cell Signal Technology
c-Src	Mouse	Monoclonal	B-12	#sc-8056	Santa Cruz Biotechnology (Santa Cruz, USA)
Flag Flag	Rabbit Mouse	Polyclonal Monoclonal		F7425 F3165	Sigma Aldrich (St Louis, USA) Sigma Aldrich
HA	Rabbit	Monoclonal	C29F4	#3724	Cell Signal Technology
HA	Mouse	Monoclonal	6E2	#2367	Cell Signal Technology
GST	Mouse	Monoclonal	B-14	sc-138 HRP	Santa Cruz Biotechnology
GAPDH	Mouse	Monoclonal	6C5	sc-32233	Santa Cruz Biotechnology
HRP-linked anti-mouse IgG	Mouse			#3724	Cell Signal Technology
HRP-linked anti-rabbit IgG	Rabbit			#7074	Cell Signal Technology

### Quantitative reverse-transcription polymerase chain reaction

Total RNA was extracted from SW480 cells cultured in a 6-well plate with or without transfection of the small peptide plasmid by using TRIzol reagent (Invitrogen) according to the manufacturer’s instructions. cDNA was synthesized using a PrimeScript™ RT Reagent Kit (Takara Biotechnology, Dalian, China). One microliter of each cDNA product was subjected to RT-qPCR by using SYBR Green PCR Master Mix (Promega) with specific primers under the following cycle conditions, denaturation at 95ºC for 10 min, followed by 40 cycles at 95ºC for 15 s and 60ºC for 1 min. The gene expression levels were calculated using the 2 ^−△△Ct^ method, and *GAPDH* was selected as the internal control. The primers used for qPCR are listed in Table 2.

**Table:** **Table 2** Sequence of primers used in the current study for qPCR

Gene	Forward sequence (5′→3′)	Reverse sequence (5′→3′)
*BRAF*	AGTACTCAGGAAAACACGACAT	CTTGGCGTGTAAGTAATCCATG
*MCM3*	GCCCGAACACTGGAAACTCTG	CCTGTGAGTCTGCCGTCTTTGGA
*E2F1*	ACGCTATGAGACCTCACTGAA	TCCTGGGTCAACCCCTCAAG
*CCND1*	ACCTGAGGAGCCCCAACAA	TCTGCTCCTGGCAGGCC
*PLK1*	GGCAACCTTTTCCTGAATGA	AATGGACCACACATCCACCT
*MYC*	GGAGGAACAAGAAGATGAGGAAGAA	AGGACCAGTGGGCTGTGAGGAG
*GAPDH*	GGAGCGAGATCCCTCCAAAAT	GGCTGTTGTCATACTTCTCATGG

### CRISPR/Cpf1-mediated *Ets1* knockout

The CRISPR/Cpf1 system was used to construct an *Ets1*-knockout cell line. Small guide RNA (5′-AGATCTGCTTGGAGTTAATAGTGGGACAATTTCTACTCTTGTAGATTGTCCACTGCCGGGGG TCTGAGGT-3′) targeting the Ets1 genome was designed and cloned into the PY30 plasmid expressing huAsCpf1 and the crRNA guide. SW480 cells were transfected with PY30-Ets1-gRNA and treated with 5 μg/mL puromycin for 3 days. Then, a single-cell dilution in a 96-well plate was used to perform clone selection, and the grown cell clones were subjected to western blot analysis to determine the knockout effect of the Ets1 protein.

### Immunofluorescence microscopy

SW480 cells were seeded on cell slides in a 24-well plate and cultured for 24 h. After the medium was decanted, the cells were washed three times with cold PBS. Then, the cells were fixed in 4% paraformaldehyde for 15 min and permeabilized in 0.5% Triton X-100 for 5 min. After the cells were washed three times with cold PBS, PBS containing 5% bovine serum albumin was used to block the samples for 1 h at room temperature. Then, 1:100 diluted indicated antibody in PBS was added to cover the cell slides and incubated overnight at 4ºC. After three washes with PBS, a 1:500 dilution of Alexa Fluor 488 anti-rabbit and a 1:1000 dilution of Alexa Fluor 647 anti-mouse antibodies (Cell Signal Technology) were added to cover the cell slides. Then, the cell nuclei were stained with DAPI for 1 h at room temperature. After five times wash with PBS, the cells on the slides were mounted by using ProLong Gold antifade reagent (Invitrogen), and images were acquired using a fluorescence microscope (Zeiss, Oberkochen, Germany).

### Co-immunoprecipitation assay

SW480 cells were cultured in 60-mm culture dishes and transfected with Ets1 and Sp1 plasmids using Lipofectamine 3000 reagent (Invitrogen). After transfection for 24 h, the cells were processed for coimmunoprecipitation by standard procedures as previously described in western blot analysis. The cell extract was used to immunoprecipitate Flag using anti-Flag magnetic beads (A36798; Thermo Fisher) according to the manufacturer’s instructions, and the immunoprecipitates were analyzed by western blot analysis using anti-HA, anti-GST and anti-Flag antibodies.

### Transfection of small inhibitory RNA

Cells were cultured to 70%‒80% confluence in 10% FBS-supplemented DMEM and transfected with Src, Ets1 or Sp1 siRNA (a pool of three target-specific 20‒25 nucleotide sequence siRNAs) using Lipofectamine 3000 reagent. A nontargeting 20‒25 nucleotide sequence siRNA was used as a negative control. Five hours after transfection, fresh medium was added to the plates. The sequence information of siRNAs used is as follows: siSp1-sense: 5′-GCAGCUACCUUGACUCCUAUU-3′; siSp1-antisense: 5′-UAGGAGUCAAGGUAGCUGCUU-3′. siEts1-sense: 5′-ACUUGCUACCAUCCCGUAC-3′; siEts1-antisense: 5′-GUACGGGAUGGUAGCAAGU-3′. siSrc-sense: 5′-CAAGAGCAAGCCCAAGGAU-3′; siSrc-antisense: 5′-AUCCUUGGGCUUGCUCUUGU-3′. siNC-sense: 5′-UUCUCCGAACGUGUCACGUTT-3′; siNC-antisense: 5′-AGCUGACACGUUCGGAGAATT-3′.

### Transwell assay

Transwell inserts were soaked in serum-free medium for 1 h in a 24-well plate. The cells were digested and the cell suspension (1.5×10 ^5^ cells/mL) was prepared with serum-free medium. Then, 300 μL cell suspension was added to the upper chamber, and serum-supplemented DMEM (or other conditioned medium and stimulating factor) was added to the lower chamber, followed by incubation for 24‒36 h in a 37ºC incubator. Transwells were removed and washed twice with PBS, fixed with 4% paraformaldehyde at room temperature for 20‒30 min and washed twice with PBS. Then, 1% crystal violet solution was added and incubated at room temperature for 0.5 h, and the surface was rinsed with running water. Cells on the upper surface were wiped off with a cotton swab, and the migrated cells were observed under a microscope and counted.

### Colony formation assay

Cells in the logarithmic growth phase were digested with trypsin, and then suspended in complete medium (basal medium+10% fetal bovine serum) and counted. For each experimental group, 400–1000 cells were inoculated into each well of a 6-well culture plate. After approximately 10 days of culture, the cells were washed with PBS and fixed with 1 mL of 4% paraformaldehyde for 30‒ 60 min in each well. One milliliter of crystal violet dye solution was added to each well, and the cells were stained for 10‒20 min. Then, cells were washed several times with PBS, images were captured with a digital camera, and the number of colonies was counted.

### Expression and purification of fusion proteins

The expression vector harboring TAT-Ets1(244-331)-GFP, TAT-Sp1(626-708)-mCherry, GST and GST-Sp1 was transformed into *E*. *BL21(DE3)*. Expression and purification of fusion proteins were performed using the following procedures. Ten milliliters of overnight culture was added to 1 L of LB media and shaken at 37ºC. When the OD _600_ reached 0.6, 0.5 mM IPTG was added to the culture with shaking at 22ºC for additional 16 h to induce protein expression. The culture was harvested by centrifugation (5600 *g*, 10 min, 4ºC) and resuspended in buffer A (50 mM phosphate, 500 mM NaCl, pH 8.0, and 0.5 mM PMSF). Then, the suspension was lysed by sonication, followed by centrifugation (14,000 *g*, 15 min, 4ºC). The supernatant was loaded onto a Ni-NTA affinity column (GE, Bethesda, USA). Purification conditions were standardized by optimizing pH, salt and imidazole concentrations. The capture column was washed with 20 mM and 40 mM imidazole sequentially, and then the fusion protein was eluted with 250 mM imidazole.

### GST pull-down assay

The GST and GST-Sp1 fusion proteins were purified as described above. GST fusion proteins were immobilized onto agarose beads for 2 h at 4ºC with rotation and then incubated for 5 h at 4ºC with the lysate of 293T cells ectopically expressing Flag-Ets1 with gentle rotation. Proteins bound to beads were washed four times with NETN buffer, and the eluted protein complexes were further subjected to western blot analysis using anti-Flag or anti-GST antibodies.

### BIFC assay

SW480 cells were cultured on cleaned coverslips in a 24-well plate at 37ºC overnight. The Ets1-Vc155 and Sp1-Vn173 and the mutant plasmids were transfected into SW480 cells using Lipofectamine 2000 (Invitrogen) according to the manufacturer’s instructions. After transfection for 24 h, the nuclear DNA of the cells was stained with Hoechst 33342, and images were captured using a microscope and representative images were selected for each sample.

### Statistical analysis

Statistical analysis was performed using SPSS 6.0 software (IBM, Armonk, USA) and GraphPad Prism 6.0. One-way analysis of variance (ANOVA) was used for comparisons among multiple groups, followed by Bonferroni’s multiple comparison tests, and Student’s *t-*tests were performed for the independent samples. Statistical significance was defined as *P*<0.05. The differential expression analysis and KEGG pathway enrichment analysis methods are detailed in the previous studies [ 35, 36] .

## Results

### Ets1 is a Sp1 binding protein

**Figure 1:**
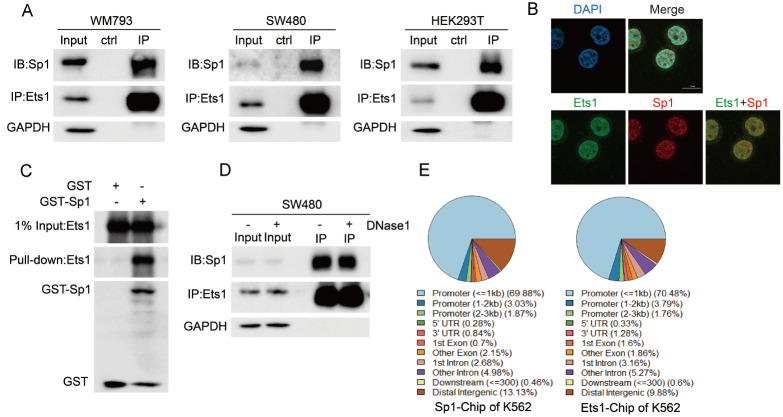
Figure 1 Identification of Ets1 as a Sp1-binding protein (A) The in vivo association of Ets1 with Sp1 was determined by using colon cancer SW480, melanoma WM793 and HEK293T cells and coimmunoprecipitation assays. Lysates from the above cells were immunoprecipitated (IP) using anti-Ets1 antibody or control IgG and sequentially immunoblotted using anti-Ets1 or anti-Sp1 antibody. (B) The localization of Sp1 and Ets1 proteins in SW480 cells was analysed by immunofluorescence assay. The cells were fixed and stained with anti-Ets1 antibody (green), anti-Sp1 antibody (red), and DAPI (blue) (scale bar= 10 μm). (C) Purified GST or GST-Sp1 fusion protein bound to agarose beads was added to the lysate of 293T cells ectopically expressing Flag-Ets1. After GST affinity purification, protein complexes were washed and detected by western blot analysis using anti-Flag or anti-GST as indicated. GST protein was used as a negative control. (D) Lysates from SW480 cells were immunoprecipitated (IP) with anti-Ets1 antibody or control IgG in the absence or presence of DNAase1 (1 unit/μL) and sequentially immunoblotted using anti-Ets1 or anti-Sp1 antibody. (E) Visualization of genomic annotation of Ets1 or Sp1-Chip data of K562 cells from GEO.

To address the interaction between Ets1 and Sp1, coimmunoprecipitation analyses were conducted in several cell lines, including two cancer cell lines, SW480 and WM793, and the human kidney cell line HEK293T ( Figure 1A). The results showed that Sp1 interacted with Ets1 in all three cell lines. In addition, an immunofluorescence assay was designed to assess the colocalization of Sp1 and Ets1 in SW480 cells. Sp1 and Ets1 were mainly located in the nucleus but less in the cytoplasm with partial colocalization ( Figure 1B). Coimmunoprecipitation and immunofluorescence studies suggested that Sp1 and Ets1 mutually interacted in SW480 cells. This result also confirmed that Ets1 acted as an Sp1 binding protein, as revealed by a GST pull-down assay *in vitro* ( Figure 1C). Because Ets1 and Sp1 are transcription factors, the interaction might be mediated via their binding at the proximal DNA locus. The interaction was further assayed after DNase I treatment of SW480 cell lysates. The addition of DNase I minimally affected the interaction between Ets1 and Sp1, suggesting that Ets1 physically interacts with Sp1 in a DNA-independent manner ( Figure 1D). These results indicated that the physical interaction of Sp1 and Ets1 might occur in the nucleus in a DNA-independent manner.

It is interesting to know if these two transcription factors colocalize in the promoter regions to regulate gene expression. Therefore, the ChIP-seq data of K562 for Ets1 and Sp1 were collected from ENCODE and GEO (ENCSR000BKQ for Ets1 and ENCSR991ELG for Sp1) to analyse whether the binding regions are enriched in the target promoters of downstream genes [37]. Both Ets1 and Sp1 are more likely to bind at the proximal promoters, within 1 kb region from the transcription start sites (TSS) ( Figure 1E and Supplementary Figure S1). The colocalization correlation between Ets1 and Sp1 indicated that the direct interaction might be involved in the regulation of gene expression to control the progression of cell activity.

### The interaction between Ets1 and Sp1 is required for migration in SW480 cells

**Figure 2:**
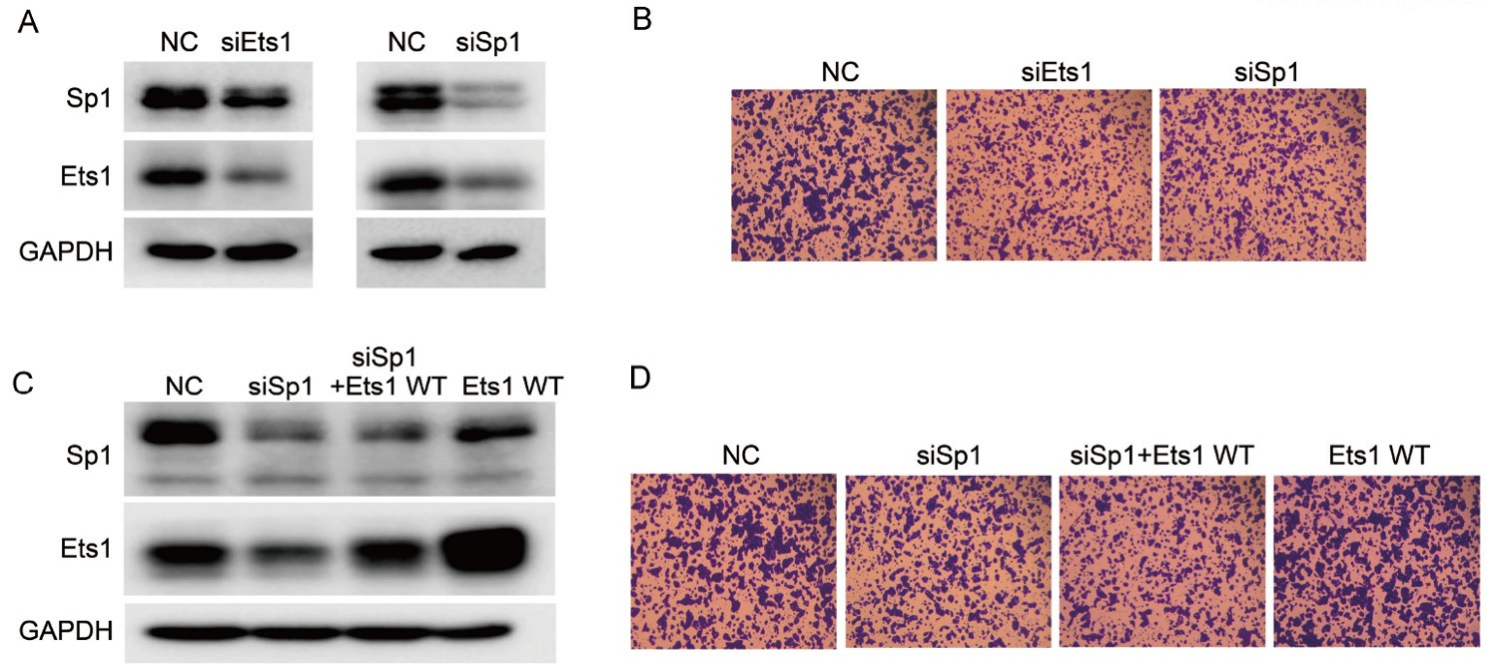
Figure 2 Sp1 is involved in Ets1 regulation of SW480 migration (A) Western blot analysis and (B) Transwell assay of SW480 cells expressing the indicated siSp1 and siEts1. Parental SW480 cells were included as a control. SW480 cells were transiently transfected with Ets1 WT-Flag plasmid or control construct after treatment with Sp1 RNAi for 20 h. Magnification fold: 10×. (C) The protein levels were determined by western blot analysis using anti-Ets1 or anti-Sp1 antibody. (D) The migration ability of SW480 cells was assayed as described in the Materials and Methods. Magnification, 10×.

Ets1 and Sp1 regulate cell progression in colorectal cancer [ 30– 32] . Downregulation of the protein level of Ets1 or Sp1 by transfecting SW480 cells with siRNA against Ets1 or Sp1 suppressed the migration of SW480 cells in comparison with the cells transfected with the control siRNA ( Figure 2A,B). It is worth noting that siSp1 downregulated the protein level of Ets1. A previous study showed that in MC3T3-E1 cells, the knockdown of *Sp1* reduced the expression of Ets1 at the transcriptional level, but the in-depth regulatory mechanism is unknown [38]. These results suggested that Sp1 played an additional role in the regulation of Ets1 expression and implied that the functions of Sp1 and Ets1 might be associated with the interaction between Sp1 and Ets1. To further address whether Ets1 functions in SW480 biotransformation independent of Sp1, Ets1 was overexpressed in siSp1 SW480 cells ( Figure 2C). Compared with NC cells, overexpression of Ets1 WT indeed promoted the migration of SW480 cells, confirming that Ets1 positively regulated the migration of SW480 cells. However,in siSp1 cells, the restoration of Ets1 expression was not able to promote cell migration ( Figure 2D). This suggested that at least SW480 migration promoted by Ets1 required the proper protein level of Sp1, possibly due to the key role of the direct interaction of Sp1 and Ets1 in the control of cell migration.

**Figure 3:**
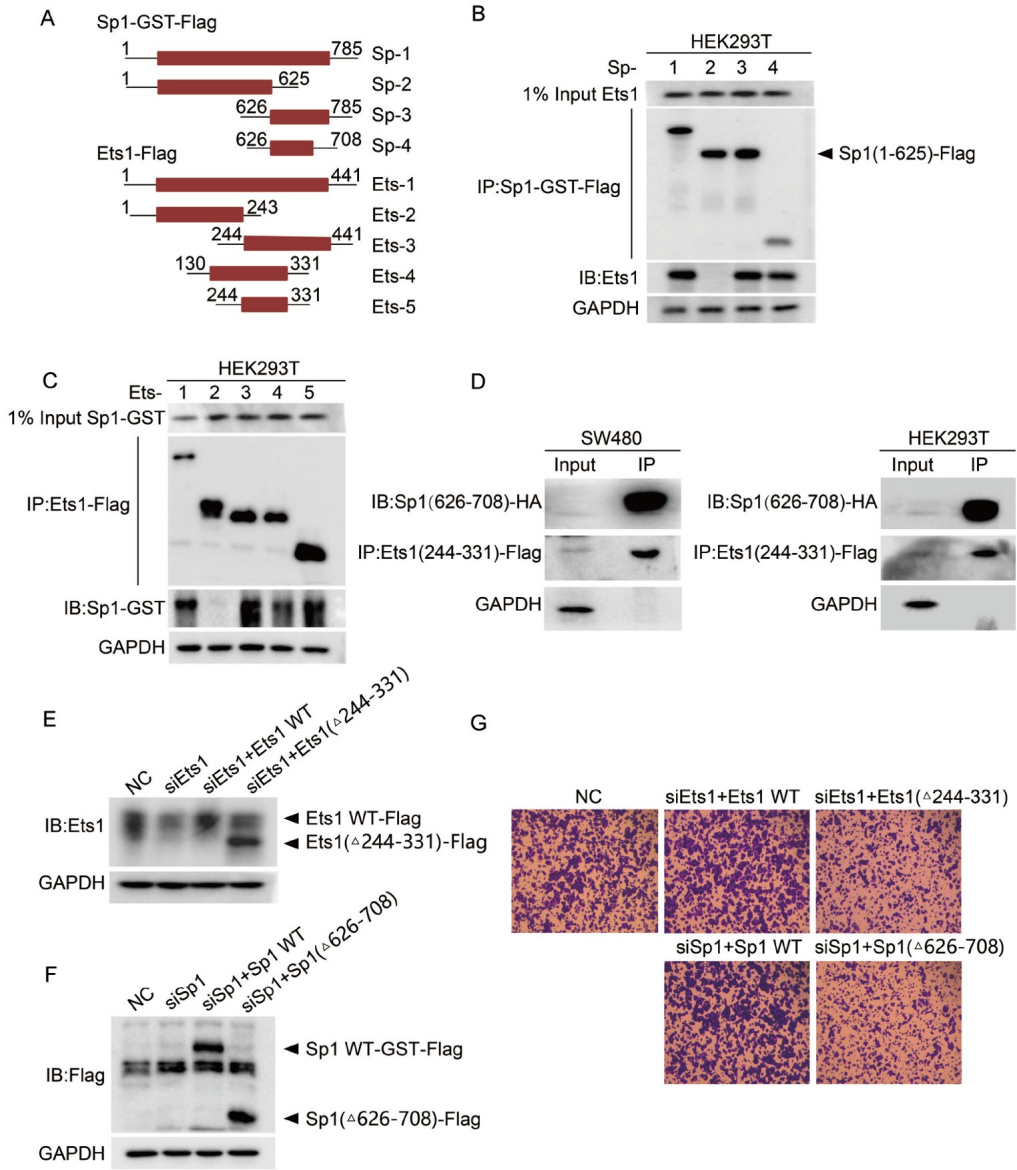
Figure 3 Ets1(244-331) interacts with Sp1(626-708), and both affect SW480 migration (A) Schematic representations of Flag-tagged Ets1 variants and GST-Flag-tagged Sp1 mutants used in a coimmunoprecipitation assay. (B) SW480 cells were cotransfected with expression constructs of GST-Flag-tagged Sp1 WT or mutants and Ets1 WT-GST and harvested after transfection for 24 h. The results of GST-Flag-tagged Sp1 IP of these cell lysates blotted using anti-Flag or anti-Ets1 antibodies are shown. (C) Consistent with the above. (D) Whole-cell lysates from colon cancer SW480 and HEK293T cells cotransfected with Sp1 and Ets1 expression vectors were immunoprecipitated (IP) using anti-Flag antibody. Ets1-Flag and Sp1-HA protein levels were determined by western blot analysis using anti-Flag or anti-HA antibody. (E,F) SW480 cells were transiently transfected with Sp1(Δ626-708)-Flag or Ets1(Δ244-331)-Flag plasmid or control after treatment with Sp1 RNAi or Ets1 RNAi for 20 h, and the protein levels were determined by western blot analysis using anti-Ets1, anti-Sp1 antibody or anti-Flag antibody. (G) The migration ability of SW480 cells was assayed as described in the Materials and Methods. Magnification, 10×.

To further confirm whether the interaction functions in SW480 migration, we studied the minimal protein domains involved in the interaction between Ets1 and Sp1. A series of plasmids encoding wild-type and various truncated forms of Ets1 or Sp1, which are tagged by Flag or GST at the C-terminus, were constructed to identify the binding regions in Sp1 and Ets1, respectively ( Figure 3A). Co-IP assays showed that the DNA binding domain of Sp1, 626-708 amino acids, is the key binding position for Ets1 ( Figure 3B). By the same strategy, the exon VII domain of Ets1, amino acids 244-331, was confirmed as the key position for Ets1 binding to Sp1 ( Figure 3C). The physical interaction between the DNA binding domain of Sp1 and the Exon VII domain of Ets1 was further confirmed in both SW480 and HEK293T cells by overexpression of both truncated peptides ( Figure 3D). Next, two segment-deletion constructs named Ets1(Δ244-331)-Flag and Sp1(Δ626-708)-Flag, encoding the domain-deletion proteins, were transfected into siEts1 or siSp1 cells, respectively. As the control in siSp1 or siEts1 cells, supplementation with Sp1 and Ets1 apparently restored the migration efficiency of SW480 cells, indicating that both Sp1 and Ets1 positively regulate the migration of SW480 cells. However, neither overexpression of Ets1(Δ244-331)-Flag nor Sp1(Δ626-708)-Flag reconstituted the migration to the extent of SW480 cells and siSp1 or siEts1 cells with the complementation of Sp1 or Ets1, respectively ( Figure 3E–G). This indicated that the two segments of Ets1(244-331) and Sp1(626-708) are necessary for Sp1 and Ets1 to positively regulate the migration process of SW480.

### Regulation of the interaction between Ets1 and Sp1 by Src to phosphorylate Ets1 Tyr283

**Figure 4:**
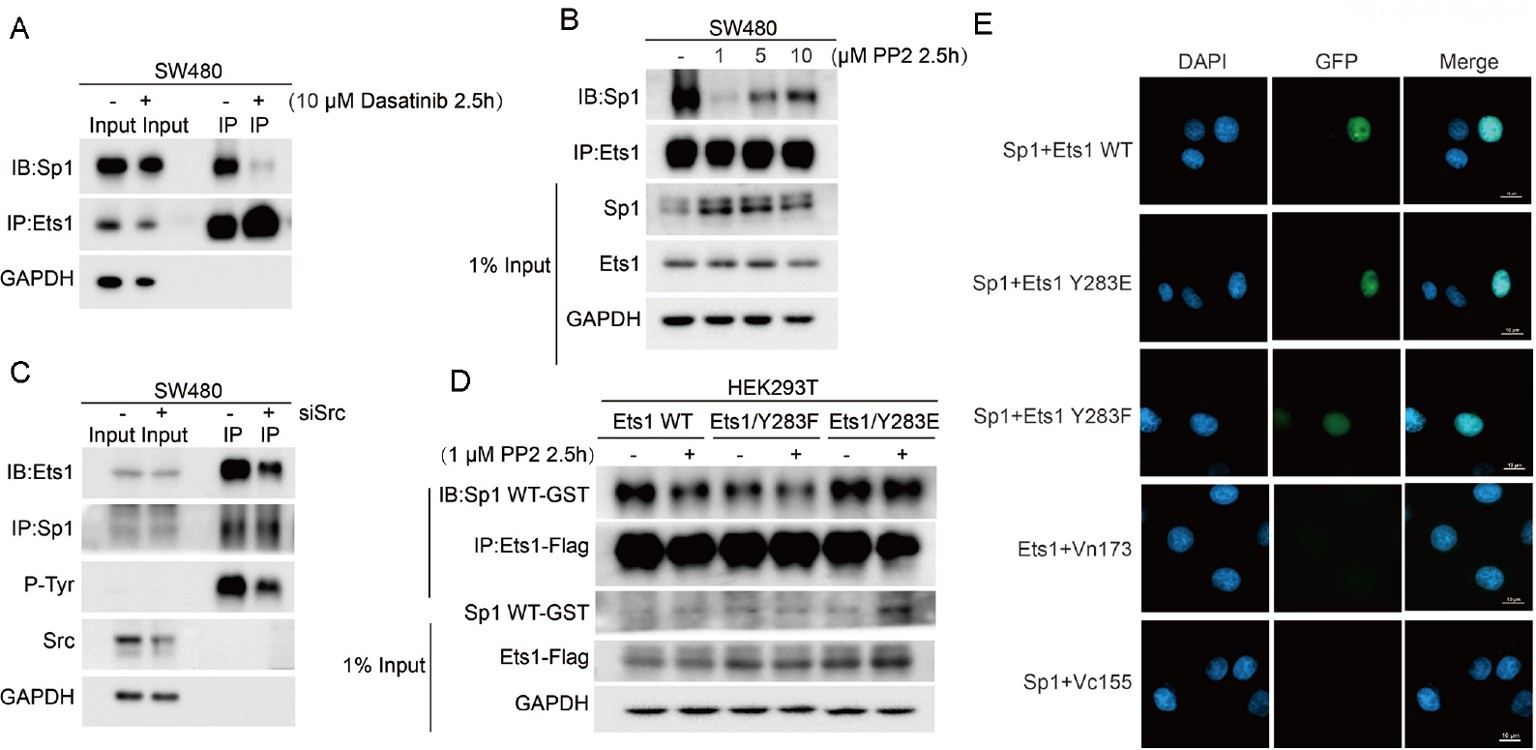
Figure 4 Src regulates the interaction between Ets1 and Sp1 via Ets1 Tyr283 (A) In vivo interaction of Ets1 with Sp1 determined using SW480 cells and a coimmunoprecipitation assay. Lysates from SW480 cells were immunoprecipitated (IP) with anti-Ets1 antibody or control IgG in the absence or presence of 10 dasatinib and (B) PP2 (1, 5 or 10 μM) for 2.5 h and sequentially immunoblotted with anti-Ets1 or anti-Sp1 antibody. (C) Lysates treated with siSrc and siNC were immunoprecipitated (IP) with anti-Sp1 antibody and subjected to western blot analysis using anti-Ets1, anti-Sp1, anti-c-Src, anti-phosphorylation-tyrosine or anti-GAPDH antibodies. GAPDH served as a loading control. (D) Western blot analysis of anti-Flag immunoprecipitates from HEK293T cells producing the indicated Flag-tagged Ets1 variants. Cells were treated with 1 μM PP2 for 2.5 h before harvest. (E) The colocalization of Ets1 with Sp1 was evaluated by BiFC assay. Ets1-Vc155, Sp1-Vn173, Ets1 WT and the mutants were transfected into SW480 cells stained with Hoechst 33342. The figures show representative fluorescent images of the indicated proteins (scale bar=10 μm).

We have proven that exon VII (amino acids 244-331) of Ets1 is essential for metastatic promotion in SW480 cells ( Figure 3). The exon VII domain of Ets1 contains a serine-rich region (SRR), which is considered to regulate the activity of Ets1 with high phosphorylation [39]. To investigate the role of Exon VII phosphorylation in the Ets1 and Sp1 interaction, SW480 cells were treated with two small molecule Src family kinase inhibitors, dasatinib and PP2. Treatment of SW480 cells with dasatinib and PP2 significantly decreased the Ets1-Sp1 interaction intensity ( Figure 4A,B) but not the Sp1 and Ets1 protein levels ( Supplementary Figure S2). This finding indicated that SRC family proteins may be upstream regulators that enhance the interaction of Ets1 with Sp1 by phosphorylating Ets1. Inactivation of Src by dasatinib and PP2 was confirmed by abrogating Src autophosphorylation at Tyr416, and dasatinib decreased Tyr283 phosphorylation of Ets1 [ 11, 40] . Next, downregulation of Src by siRNA decreased Ets1 tyrosine phosphorylation with respect to Sp1 reciprocal binding in coimmunoprecipitation experiments ( Figure 4C). These data showed that the cooperation of Ets1 with Sp1 is affected by Src kinase and tyrosine phosphorylation of Ets1.

To address the importance of Tyr283 phosphorylation in the control of the Ets1-Sp1 interaction by Src family kinases, SW480 cells were transfected with constructs encoding Ets1 WT-Flag, Ets1 Tyr283F-Flag (Ets1/Y283F), or Ets1 Tyr283E-Flag (Ets1/Y283E) in the presence of PP2. Treatment with PP2 decreased Sp1 binding with Ets1 WT in cells ( Figure 4D). The binding of Sp1 to the Tyr283F variant was apparently decreased in comparison with Ets1 WT but was not further affected by PP2 treatment ( Figure 4D). Additionally, the Tyr283E variant was able to bind to Sp1 more strongly than Ets1 Tyr283F but was not affected by treatment with PP2 ( Figure 4D). These results revealed that phosphorylation of the Ets1 Tyr283 site regulates the binding affinity of Ets1 to Sp1. The bimolecular fluorescence complementation (BiFC) assay was further designed to assess the regulation of the Ets1 Tyr283 site for the interaction between Sp1 and Ets1. The plasmids coding Ets1-Vc155, Ets1-Y283E-Vc155 or Ets1-Y283F-Vc155 were cotransfected with the plasmid coding Sp1-Vn173 into SW480 cells. Weaker fluorescence was observed in SW480 cells cotransfected with Sp1 and Ets1 Y283F than in SW480 cells cotransfected with Sp1 and Ets1 Y283E or with Sp1 and Ets1 WT ( Figure 4E). The statistical analyses are shown in Supplementary Figure S3 **.** These results indicated that phosphorylation of Ets1 at the Tyr283 site facilitates the interaction between Sp1 and Ets1.

### Src and Ets1 Tyr283 phosphorylation affects SW480 cell migration

**Figure 5:**
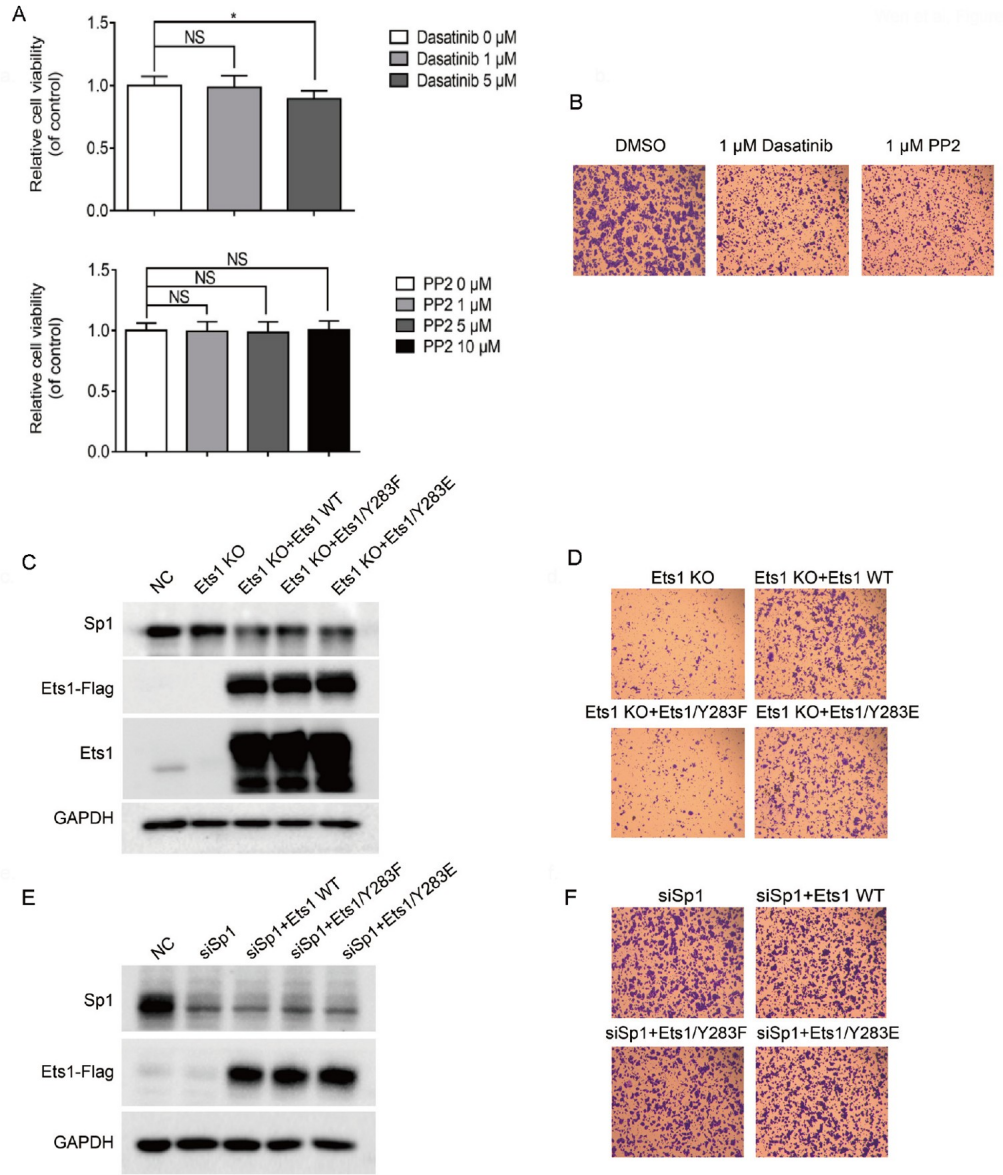
Figure 5 The Src/Ets1 pathway affects SW480 migration (A) CCK-8 and (B) Transwell assays of SW480 cells treated with dasatinib and PP2 for 24 h. Magnification fold: 10×. (C,E) Western blot analysis and (D,F) Transwell assay of SW480 cells expressing Ets1 WT, Tyr283F and Tyr283E-Flag after the indicated Sp1 siRNA treatment for 20 h or in Ets1-knockout cells. Parental SW480 cells were included as a control. Magnification fold: 10×. Data are shown as the mean±SEM of at least three independent experiments. Statistical significance was defined as * P<0.05. NS means no significance.

We confirmed that inhibition of Src abrogated the interaction between Ets1 and Sp1 by decreasing Ets1 Tyr283 phosphorylation. To determine the role of the Src/Ets1 pathway in SW480 cell migration, SW480 cells were treated with dasatinib and PP2. The results showed that migration was apparently decreased within 24 h and at 1 μM, which did not affect cell viability ( Figure 5A,B). Next, to study whether Tyr283 phosphorylation is required to promote metastatic ability, *Ets1* knockout was constructed in SW480 cells by using the CRISPR/Cpf1 system. In *Ets1*-KO cells, overexpression of both Ets1 WT and Tyr283E variant was able to promote metastatic SW480 compared with Tyr 283 F ( Figure 5C,D), suggesting that Ets1 Tyr283 site phosphorylation is essential for promoting SW480 migration, but whether Ets1 Tyr283 phosphorylation plays an important role in SW480 metastasis in an Sp1-dependent manner remains to be further addressed. The metastasis of siSp1 SW480 was observed when Ets1 and its variants thereof were overexpressed. As shown in Figure 5E,F, overexpression of both Ets1 WT and the variants did not promote the migration in siSp1 cells compared with the control, siSp1 cells. In summary, the inhibition of the Src/Ets1 pathway attenuated the migration of SW480 cells due to the control of the Ets1-Sp1 interaction by the phosphorylation of Tyr283.

### Treatment with Ets1(244-331) and Sp1(626-708) inhibits SW480 metastasis

**Figure 6:**
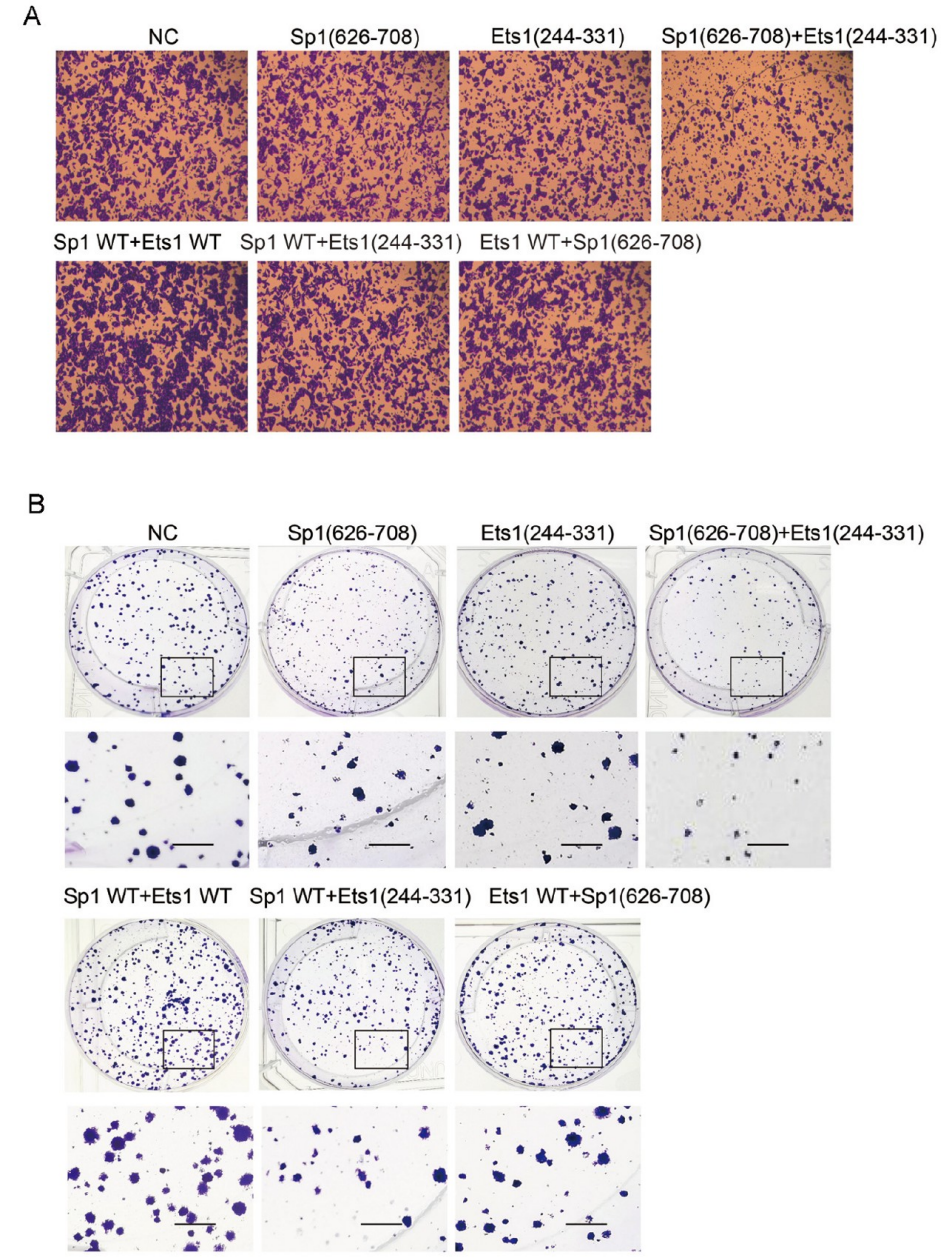
Figure 6 The interaction between Ets1 and Sp1 affects the transformation of SW480 cells (A) Transwell and (B) colony formation assays of SW480 cells transfected with indicated expression plasmids of Sp1 and Ets1. Magnification, 10×(Transwell) and 5 mm (colony formation).

According to the data, overexpression of either or both Ets1 and Sp1 promoted SW480 metastasis, probably due to the Ets1 and Sp1 interaction in promoting cell migration ( Figure 6A and Supplementary Figure S4A‒C). It is worth examining whether Ets1(244-331) and Sp1(626-708) function as potential antitumour agents to inhibit SW480 migration depending on the competitive interrogation of the Ets1-Sp1 native interaction. Co-transfections with different combinations of Ets1(244-331) or Sp1(626-708) were implemented in SW480 cells. The results showed that co-overexpression of Ets1 or Sp1 mutants did not promote but inhibited cell migration, suggesting that the minimal binding domain might serve as a competitor to interrupt the endogenous interaction between Ets1 and Sp1 in the metastasis of SW480 cells. Overexpression of Sp1(626-708) or Ets1(244-331) inhibited the migration ability and colony formation of SW480 cells, and cotransfection with Sp1(626-708) and Ets1(244-331) further reduced cell migration and colony formation capability compared to transfection with the mutants ( Figure 6A,B). The statistical analyses are shown in Supplementary Figure S5. Additionally, overexpression of Sp1(626-708) and Ets1(244-331) did not affect the endogenous Sp1 and Ets1 protein levels ( Supplementary Figure S6). This suggested that the two peptides harboring interaction domains may serve as potential therapeutic inhibitors for carcinogenesis controlled by the Ets1 and Sp1 interaction.

**Figure 7:**
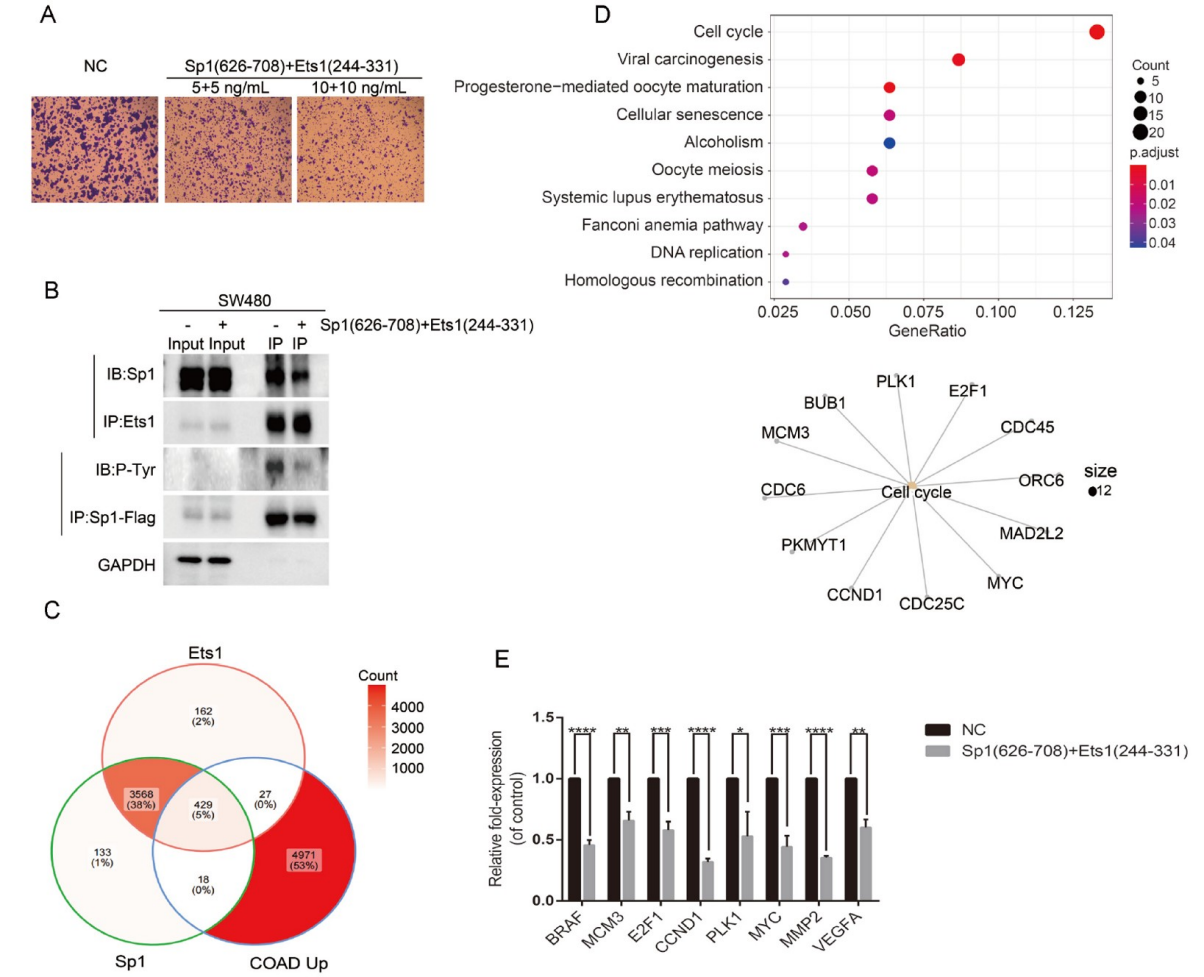
Figure 7 The two shortest interaction domains might be used as a treatment strategy for SW480 tumor (A) Transwell assay of SW480 cells expressing Sp1(626-708) and Ets1(244-331) fusion protein with increasing concentration. Magnification, 10×. (B) Whole-cell lysates from colon cancer SW480 cells cotransfected with Sp1(626-708) and Ets1(244-331) expression vectors were immunoprecipitated (IP) with anti-Flag and anti-Ets1 antibodies. Sp1 WT-Flag, Ets1, Sp1 and P-Tyr protein levels were determined by western blot analysis using anti-Flag, Ets1, Sp1, and P-Tyr antibodies. (C) Venn diagram of genes that bind to Ets1 and Sp1 and are upregulated in the primary tumor compared with normal colon tissue. (D) KEGG pathway enrichment analysis of target genes, P<0.05. (E) Expressions of target genes were detected by RT‒PCR analysis. * P<0.05, ** P<0.01, *** P<0.001,**** P<0.0001

To further evaluate the potential inhibitory effects of the two peptides on SW480 cell migration, Ets1(244-331) and Sp1(626-708) were fused with Cell Penetrating Peptide (YGRKKRRQRRR), expressed in *E*. *coli* BL21DE3, and purified *in vitro*. The two purified fusion proteins were directly added to the cell culture for SW480 metastasis measurements. Additionally, we purified the GFP-tagged and mCherry-tagged fusion proteins and detected their transmembrane features by fluorescence measurement. Apparent fluorescence was observed in SW480 cells after 6 h of treatment with GFP- and mCherry-tagged proteins, and the cell morphology and growth conditions did not change significantly compared with the control ( Supplementary Figure S7A,B). This suggested that the fusion proteins have good membrane permeability with low toxicity. When SW480 cells were treated with 5 or 10 ng/mL fusion proteins, cell migration was proportionally decreased with increasing fusion protein concentration ( Figure 7A and Supplementary Figure S8). Furthermore, to explore whether the two peptides attenuate SW480 cell migration by affecting the interaction between Sp1 and Ets1, the interaction of Sp1 with Ets1 and the tyrosine phosphorylation level of Ets1 were detected by Co-IP and western blot analysis. The results showed that both the interaction of Sp1 with Ets1 and the level of tyrosine phosphorylation of Ets1 were reduced by the cotransfection with Ets1(244-331) and Sp1(626-708) plasmids in SW480 cells ( Figure 7B and Supplementary Figure S9). In summary, the two peptides harboring interaction domains attenuated SW480 cell migration by reducing the endogenous interaction of Sp1 with Ets1 and the level of tyrosine phosphorylation of Ets1.

Furthermore, by analysis of the Chip-Seq data of Sp1 and Ets1 and TCGA colon cancer project data, where the 4971 genes with significant differences in expression at the primary site compared to normal tissues, 429 genes were identified as significantly different in expression levels ( Figure 7C). KEGG pathway enrichment analysis enriched a total of 12 genes ( *P*<0.05) related to the cell cycle process ( Figure 7D). Six cell cycle-related genes ( *BRAF*, *MCM3*, *E2F1*, *CCND1*, *PLK1* and *MYC*) were chosen to examine their mRNA levels for the expression difference in two fusion protein-treated SW480 cells and untreated control cells. In addition, a series of studies have shown that the downregulation of these genes can inhibit the migration and invasion ability of colon cancer or other cancer cells [ 41– 53] . The mRNA levels of the above genes were significantly downregulated in SW480 cells treated with Ets1(244-331) and Sp1(626-708) ( Figure 7E and Supplementary Figure S9). We also examined the expression of the metastasis-specific genes *MMP2* and *VEGFA* in SW480 cells after the addition of the interaction segments of Est1 and Sp1. The ChIP-seq data revealed that both Sp1 and Ets1 bind to proximal promoters, such as *PLK1* and *VEGFA* ( Supplementary Figure S10). In addition, the mRNA levels of *MMP2* and *VEGFA* were significantly downregulated in SW480 cells treated with Ets1(244-331) and Sp1(626-708) ( Figure 7E). In summary, these findings indicated that two peptides, Ets1(244-331) and Sp1(626-708), might be applied as potential treatments for the inhibition of SW480 metastasis.

## Discussion

Ets1 is well characterized as a proto-oncogene transcription factor due to its high expression related to poor prognosis in cancers, including colorectal cancer, and the transcriptional regulation of genes related to cell migration, invasion, and replication [52]. Targeting Ets1 in cancer treatment has been proposed, and the myriad ways by which downregulation of Ets1 protein inhibits tumor progression. For example, Src kinase, the upstream activator of the signal cascade, stabilizes the Ets1 protein through posttranslational modification [11]. Therefore, dasatinib, an inhibitor of Src kinase activity, can effectively reduce Ets1 protein and significantly suppress the development of metastatic and invasive cisplatin-resistant head and neck squamous cell carcinoma [16]. Similarly, its knockdown can effectively inhibit tumor progression and growth in melanoma and gastric and breast cancer [ 11, 12, 55] .

However, as a transcription factor, Ets1 interacts with other regulatory factors and plays an important role by altering gene transcription in the control of cancer-relevant processes. This study explains the functional interaction between Ets1 and other regulatory factors in the process of migration.

Our results showed that Ets1 directly interacts with Sp1 independent of DNA in SW480 colon cancer cells, and knockdown of *Sp1* caused a decrease in the expression level of Ets1 protein, in agreement with a previous study that showed that in MC3T3-E1 cells, the knockdown of *Sp1* reduced the expression of Ets1 at the transcriptional level [38]. Furthermore, it has been reported that Ets1 and Sp1 can cooperatively modulate oncogene expression [ 54, 56– 58] . Therefore, it is interesting to explore the function of the interaction between Sp1 and Ets1 in cell metastasis.

Unlike previous results, we found that SW480 cell metastasis and colony formation in SW480 cells depended on the direct interaction between Ets1 and Sp1 rather than Ets1 alone. Furthermore, we addressed the interaction domains, which are the segment at Sp1(626-708) amino acids and the segment at Ets1(244-331) amino acids. Interestingly, the two truncated peptides strongly inhibited the colony formation and migration of SW480 cells.

Notably, Ets1(244-331) amino acids are highly phosphorylated in the serine-rich region (SRR) [39], which includes the Tyr283 residue that regulates Ets1 stability. It has been reported that Ets1 Tyr283 phosphorylation stabilizes the Ets1 protein via Src kinase [11]. Here, we found that Src regulates the Sp1-Ets1 interaction through phosphorylation at Ets1 Tyr283, suggesting that the Src/Ets1 pathway not only affects the level of the Ets1 protein but also promotes its interaction with Sp1. In addition, we also demonstrated that phosphorylation of Ets1 at Tyr283 promotes SW480 migration dependent on the protein level of Sp1 in the cells.

In conclusion, we provide a novel view on the role of Ets1 as a proto-oncoprotein in the tumor process, which suggests that metastasis and colony formation in SW480 cells depend on the direct interaction between Ets1 and Sp1 instead of high Ets1 expression. Furthermore, our data provide new insights into the Ets1 and Sp1 interaction in SW480 cells as an antitumour target.

## Supporting information

584Supplemental_Information-wen-20220930

## Supplementary Data

Supplementary data is available at *Acta Biochimica et Biophysica Sinica* online.
