# Supplementary material for: Phosphorylation-mediated interaction between human E26 transcription factor 1 and specific protein 1 is required for tumor cell migration: Ets1 and Sp1 interact to promote SW480 migration
Source: Acta Biochim Biophys Sin (Shanghai). 2022 Oct 27;54(10):1441–52. doi: 10.3724/abbs.2022148 (PMC9828152; doi:10.3724/abbs.2022148)
Supplement: 584Supplemental_Information-wen-20220930 [file 584Supplemental_Information-wen-20220930.pdf]

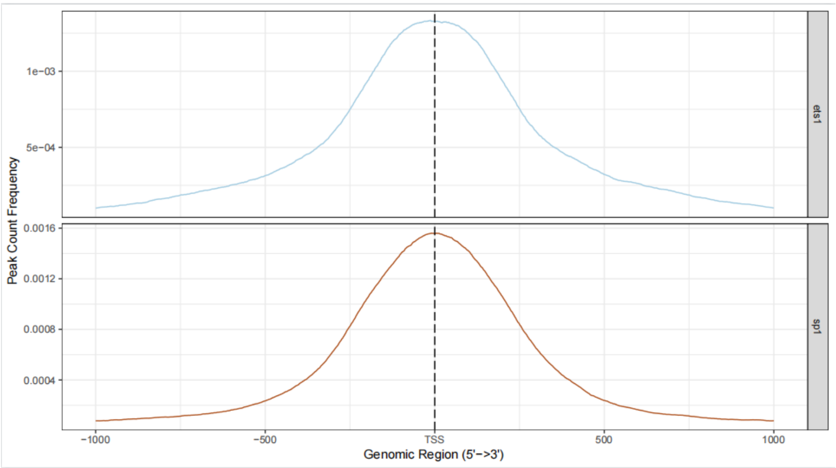

Supplementary Figure S1. Ets1 and Sp1 mostly bind to the proximal promoter regions The GEO ChIP-seq data of Ets1 and Sp1 were analyzed to check the binding regions enriched in the target promoters of downstream genes.

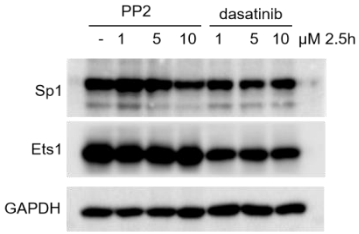

Supplementary Figure S2. PP2 did not affect the protein levels of Sp1 and Ets1 SW480 cells were treated with different concentrations of Dasatinib or PP2 for 2.5 h, and the protein levels were determined by western blot analysis using anti-Ets1 or anti-Sp1 antibody.

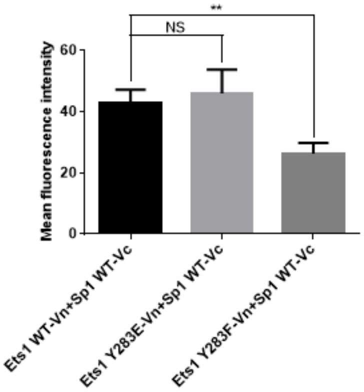

Supplementary Figure S3. Statistical analysis of the effects of BIFC assay The data (mean values ± standard deviations of three independent experiments) are presented as the fold-change with respect to the control group. Significant differences from the positive control and control groups are indicated by *P < 0.05, **P < 0.01 and ***P < 0.001.

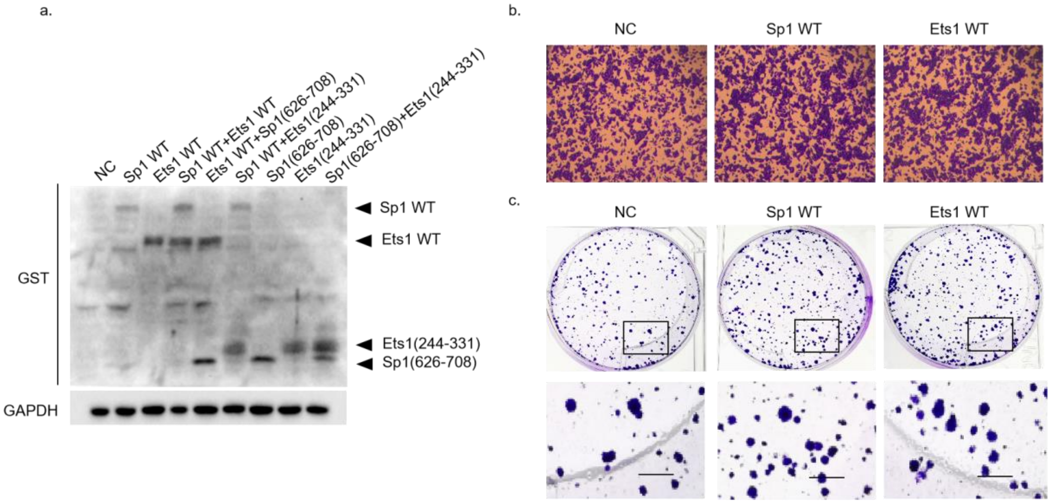

Supplementary Figure S4. Sp1 and Ets1 promote SW480 cells migration and colony formation (a) SW480 cells were transfected with WT or variants plasmid of Sp1 and Ets1, and GST-tagged Ets1 and Sp1 protein levels were determined by western blot analysis using anti-GST antibody. (b,c) Transwell and colony formation assays of SW480 cells transfected with Sp1 and Ets1 WT plasmid.

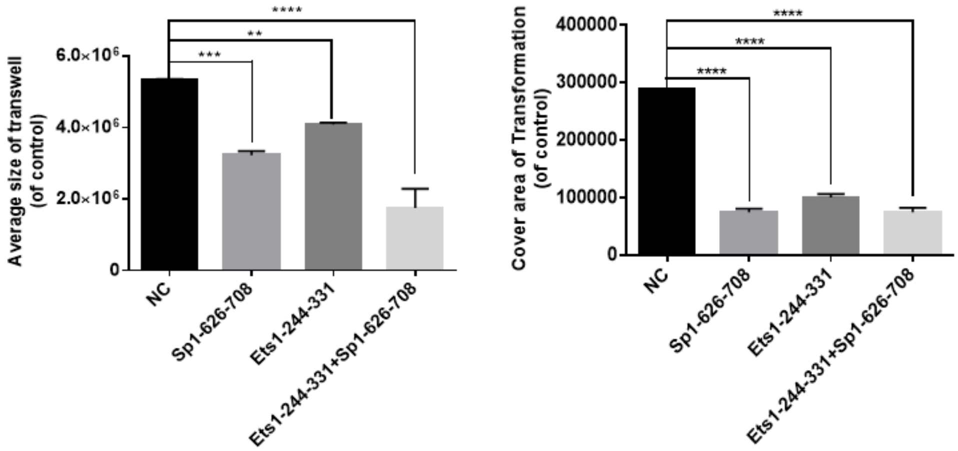

Supplementary Figure S5. Statistical analysis of the effects of Sp1 (626-708) and Ets1 (244-331) on the migration and colony formation of SW480 cells Statistical analysis of migration (left) and colony formation (right). The data (mean values ± standard deviations of three independent experiments) are presented as the fold-change with respect to the control group. Significant differences from the positive control and control groups are indicated by *P < 0.05, **P < 0.01 and ***P < 0.001.

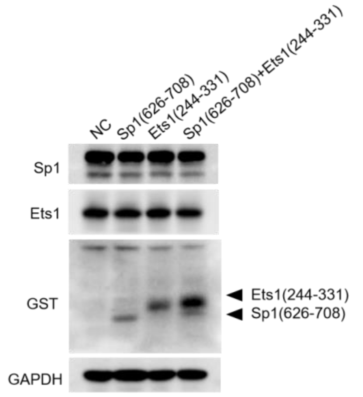

Supplementary Figure S6. Sp1 (626-708) and Ets1 (244-331) did not affect the endogenous Sp1 and Ets1 protein levels SW480 cells were transfected with GST-tagged Sp1 (626-708) and Ets1 (244-331), and the proteins level were determined by western blot analysis using anti-GST, anti-Ets1 and anti-Sp1 antibodies.

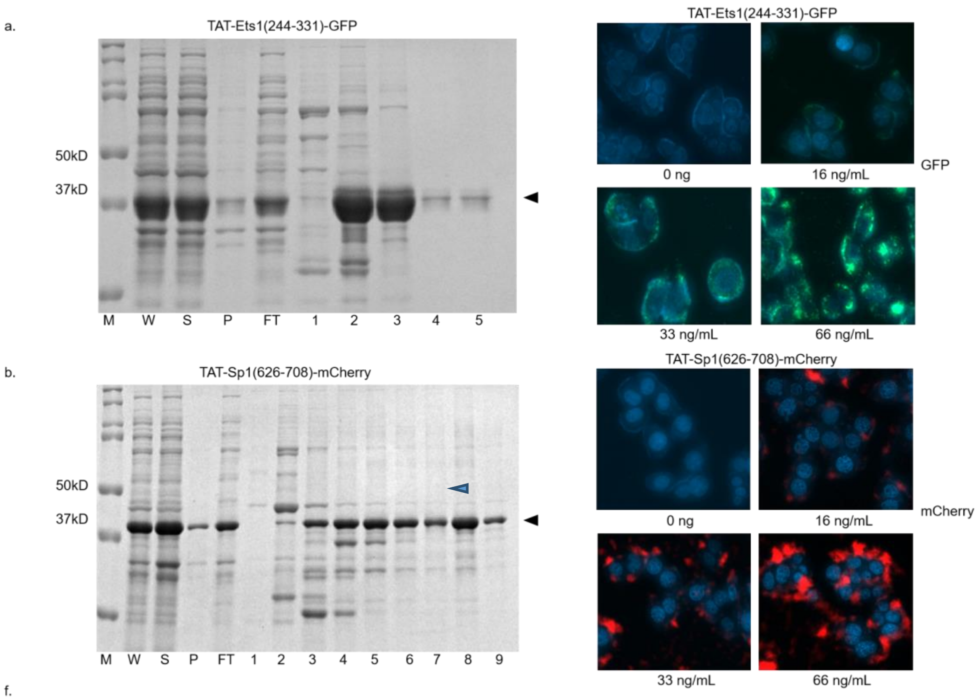

Supplementary Figure S7. Purification and validation of TAT-Ets1 (244-331)-GFP and TAT-Sp1 (626-708)-mCherry protein SDS-PAGE and immunofluorescence assay of (a) AT-Ets1 (244-331)-GFP and (b) AT-Sp1 (626-708)-mCherry fusion proteins.

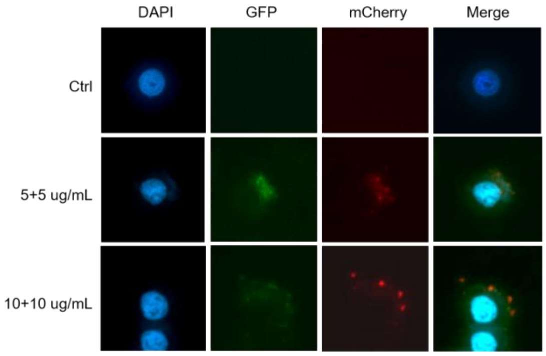

Supplementary Figure S8. The fusion proteins have good membrane permeability with low toxicity Imunofluorescence assay of SW480 cells treated with increasing concentrations of TAT-Ets1 (244-331)-GFP and TAT-Sp1 (626-708)-mCherry fusion proteins.

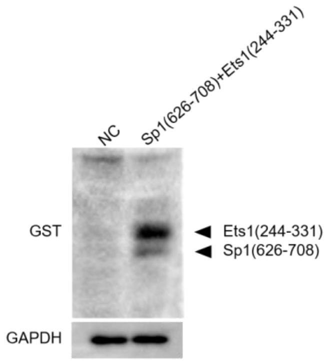

Supplementary Figure S9. Expression of Sp1 (626-708) and Ets1 (244-331) in SW480 cells SW480 cells were transfected with GST-tagged Sp1 (626-708) and Ets1 (244-331), and the protein levels were determined by western blot analysis using anti-GST antibody.

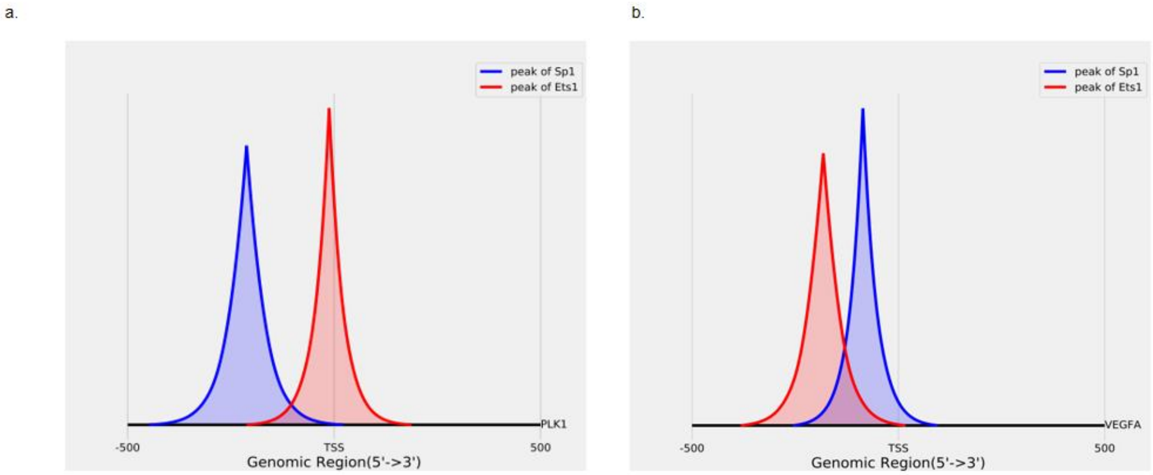

Supplementary Figure S10. The overlap binding patterns of Ets1 and Sp1 proteins for *PLK1* and *EGFA* genes (a,b) The GEO ChIP-seq data of Ets1 and Sp1 were analyzed to check the binding regions enriched in *PLK1* and *VEGFA* genes.
